# Supplementary material for: Long-Term Tetrabromobisphenol A Exposure Induces Gut Microbiota Imbalance and Metabolic Disorders via the Peroxisome Proliferator-Activated Receptor Signaling Pathway in the Regenerated Gut of Apostichopus japonicus
Source: Biology (Basel). 2023 Oct 25;12(11):1365. doi: 10.3390/biology12111365 (PMC10669644; doi:10.3390/biology12111365)
Supplement: Supplementary file 1 [file biology-12-01365-s001.zip › biology-2611766-supplementary.pdf]

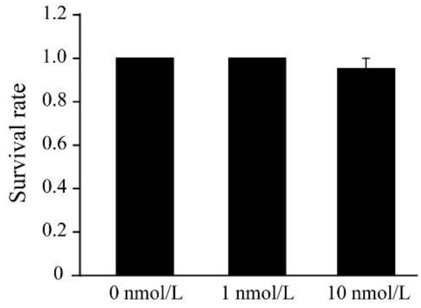

Figure S1: Survival of A. japonicus after TBBPA challenge.

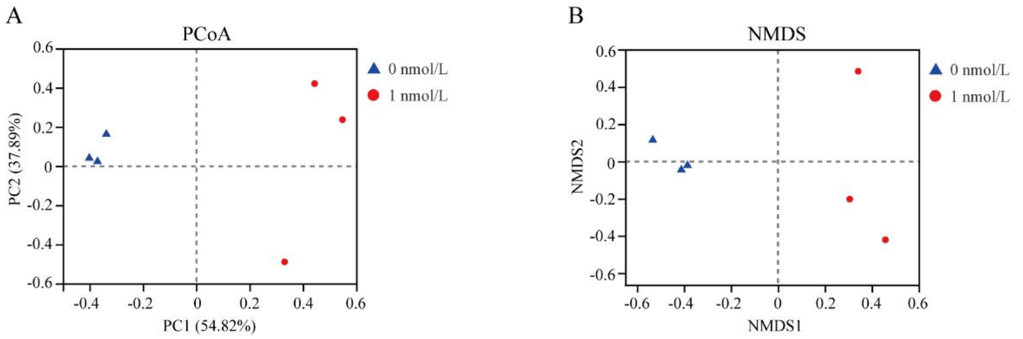

Figure S2. Beta diversity analysis was used to assess differences in microbial composition in the regenerated intestine of A. japonicus after TBBPA challenge.

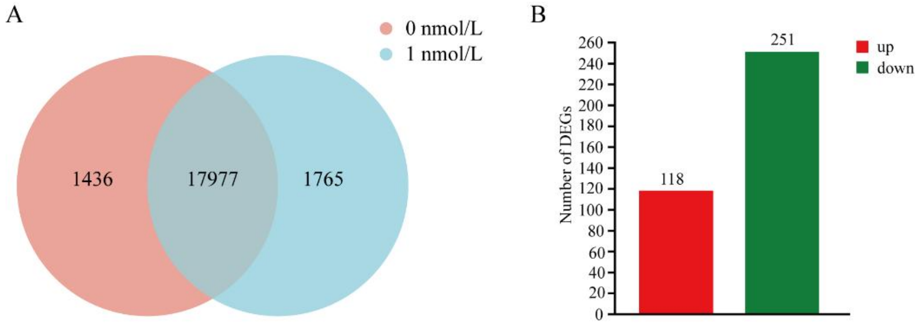

Figure S3. The number of annotated genes and differentially expressed genes after the TBBPA challenge.

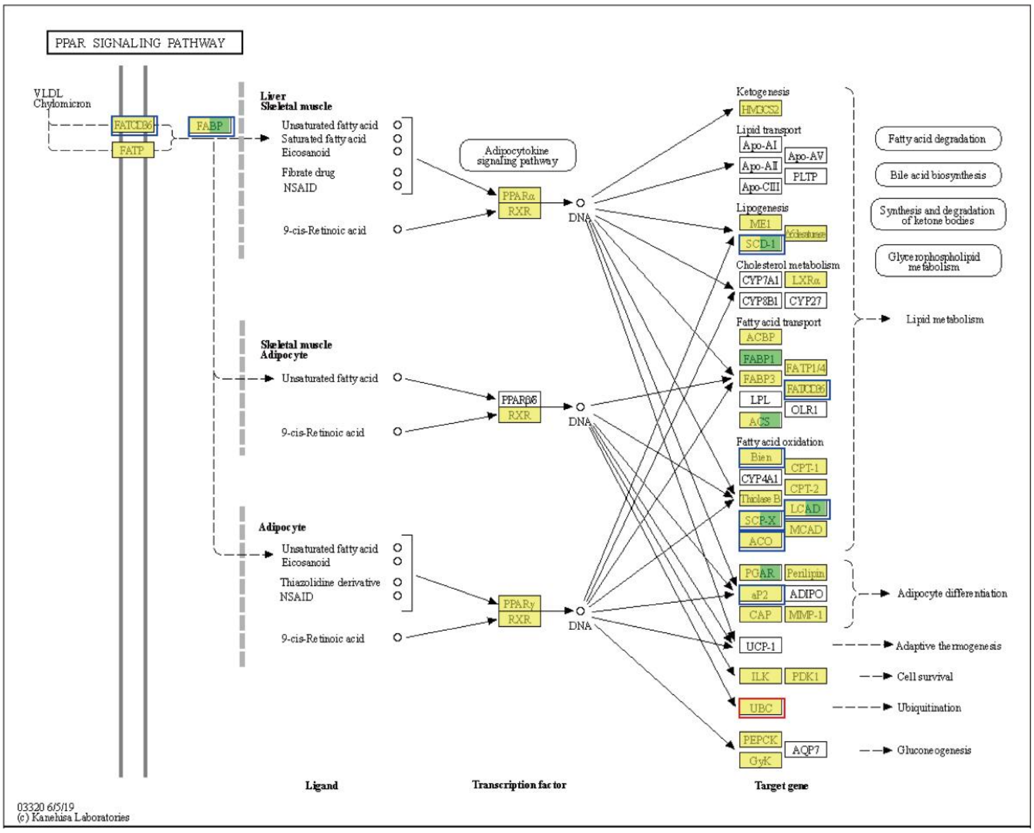

Figure S4. Overview of PPAR signaling pathway by KEGG enrichment analysis.

Table S1. Summary of RNAseq data.

Sample ID	Read Number	Base Number	GC Content %	Q30	Mapped Ratio
0 nmol/L-1	43996224	6523770887	39.14	91.63	0.9729
0 nmol/L-2	42730754	6209159044	40.76	93.46	0.9731
0 nmol/L-3	42713958	6221981364	40.05	93.24	0.9704
1 nmol/L-1	40410944	5976185129	39.94	90.14	0.9739
1 nmol/L-2	42266526	6260927283	39.82	92.45	0.9742
1 nmol/L-3	42567906	6292766917	37.93	92.28	0.9722

Table S2. The primes were used in this study.

Prime name	Sequences (5'--3')
β-actin-F	AAGGTTATGCTCTTCCTCACGCT
β-actin-R	GATGTCACGGACGATTTCACG
SCD3-F	AGGTGGTGGTATTGATTGC
SCD3-R	GGAGGTAGGTCGGAAGAA
EHHADH-F	GATGAGTTGGCAGATATG
EHHADH-R	GTCTTCCTCTTACATTCTC
SCP2-F	CATTAGAATCCGACCAGTAGAG
SCP2-R	TCGCTGTCTGTCATCGTA
FABP-F	GCGATTGTTGGTGCTCTT

FABP-R	CGTCTGCCTTGAACTTCTC
UBB-F	TGGTGGACAAGGAAGGTAT
UBB-R	AGACGAAGGACAAGATGGA
SCD2-F	ACAGCACAGAATGAACCGAAG
SCD2-R	AGGATAGTGACGCAATGATAGC
SCD1-F	GCCTGTCCTGTCAGAACTCC
SCD1-R	TTACCCAAAGCCAACCCAT
FATCD36-F	TGTGCCAGTGCTACATTACG
FATCD36-R	CATGCCTTCAACCATATCCA
ACADL-F	TAGGAGCCGCTGGTTTCTT
ACADL-R	TCTCCTTCTGTTCCTGGGTG
FASN-F	AATGGTCTGGTGCGTCTTA
FASN-R	TATGGTGATGGCGTTGGA
